# Total Lesion Glycolysis Estimated by a Radiomics Model From CT Image Alone

**DOI:** 10.3389/fonc.2021.664346

**Published:** 2021-06-17

**Authors:** Hongwei Si, Xinzhong Hao, Lianyu Zhang, Xiaokai Xu, Jianzhong Cao, Ping Wu, Li Li, Zhifang Wu, Shengyang Zhang, Sijin Li

**Affiliations:** ^1^ Department of Nuclear Medicine, The First Affiliated Hospital of Anhui Medical University, Hefei, China; ^2^ Nuclear Medicine, The First Affiliated Hospital of Shanxi Medical University, Collaborative Innovation Center for Molecular Imaging of Precision Medicine, Shanxi Medical University, Taiyuan, China; ^3^ Department of Diagnostic Imaging, National Cancer Center/ Cancer Hospital, Chinese Academy of Medical Sciences and Peking Union Medical College, Beijing, China; ^4^ Department of Radiation Oncology, The Cancer Hospital of Shanxi Province, Taiyuan, China

**Keywords:** radiomics, standard uptake value, positron emission tomography, computed tomography, total lesion glycolysis

## Abstract

**Purpose:**

In this study, total lesion glycolysis (TLG) on positron emission tomography images was estimated by a trained and validated CT radiomics model, and its prognostic ability was explored among lung cancer (LC) and esophageal cancer patients (EC).

**Methods:**

Using the identical features between the combined and thin-section CT, the estimation model of SUVsum (summed standard uptake value) was trained from the lymph nodes (LNs) of LC patients (n = 1239). Besides LNs of LC patients from other centers, the validation cohorts also included LNs and primary tumors of LC/EC from the same center. After calculating TLG (accumulated SUVsum of each individual) based on the model, the prognostic ability of the estimated and measured values was compared and analyzed.

**Results:**

In the training cohort, the model of 3 features was trained by the deep learning and linear regression method. It performed well in all validation cohorts (n = 5), and a linear regression could correct the bias from different scanners. Additionally, the absolute biases of the model were not significantly affected by the evaluated factors whether they included LN metastasis or not. Between the estimated natural logarithm of TLG (elnTLG) and the measured values (mlnTLG), significant difference existed among both LC (n = 137, bias = 0.510 ± 0.519, r = 0.956, P<0.001) and EC patients (n = 56, bias = 0.251± 0.463, r = 0.934, P<0.001). However, for both cancers, the overall shapes of the curves of hazard ratio (HR) against elnTLG or mlnTLG were quite alike.

**Conclusion:**

Total lesion glycolysis can be estimated by three CT features with particular coefficients for different scanners, and it similar to the measured values in predicting the outcome of cancer patients.

## Introduction

Radiomics has gained attention recently due to the quantitative information extracted from medical images and derived features, which are associated with cancer phenotype, patient outcome, treatment response, and other classification data (1, 2). However, studies are seldom designed for estimating *in vivo* quantitative data, which might be predicted or prognostic biomarkers. Therefore, this study was designed to explore its feasibility.

Radioactive 18F labeled Fluorodeoxyglucose (18F-FDG) is an analog of glucose, and its distribution *in vivo* can be imaged by a positron emission tomography scanner (PET). Because the resolution of PET images is lower than other medical imaging modalities, an additional CT (combined CT) scan is conventionally acquired to accurately locate the abnormal uptake of tracer. Therefore, commercial scanners are simultaneously equipped with PET and CT scanners, and the images are successively acquired. In PET/CT image analysis, standard uptake value (SUV) is the most frequently used semi-quantitative parameter for measuring FDG uptake and is a unitless value of a single pixel in a lesion. There are several types of SUV, for example, maximal SUV (SUVmax) and sum SUV (SUVsum) means the maximum and total value of a primary tumor (or a lymph node), respectively (3–5).

SUVmax of the primary tumor is commonly used in various PET/CT reports, and is related to the prognosis of myeloma, lymphoma, and other cancers (3–5); however, the correlations could be misled by the measurement of SUVmax of a single pixel (6). Therefore, the total lesion glycolysis (TLG), which includes the accumulated SUVsum of lesions in a patient has been proposed as an index of tumor load and proved to be a much stronger surrogate marker than SUVmax (7, 8).

In this study, we trained and validated a CT radiomic model to estimate the SUVsum of each lymph node or primary tumor on PET images. The prognostic ability of the estimated TLG was then evaluated and compared to the measured value.

## Materials and Methods

### Patients

From January 2015 to December 2019, pathologically confirmed lung cancer (LC) patients were screened from the First Affiliated Hospital of Shanxi Medical University (SMU), the First Affiliated Hospital of Anhui Medical University (AMU), and the RIDER Lung PET-CT dataset (9) of The Cancer Imaging Archive (TICA) (10). Additionally, another group of esophagus cancer (EC) from SMU was also included as one of the validation cohorts. The study design was approved by the ethics committees at the First Affiliated Hospital of Shanxi Medical University and the First Affiliated Hospital of Anhui Medical University, and was registered at ClinicalTrials.gov (NCT03648151). For a retrospective study, the two review boards waived the requirement for informed consent.

Eligible patients had accepted PET/CT scans (PET and combined CT), and at least one LN (short-axis diameter > 3mm) was visible on CT images identified by two experienced radiologists. For the lung cancer patients from SMU (SMU LC), besides PET/CT images, an additional thin-section chest CT scan was acquired. The excluded patients had a history of diabetes, chronic heart diseases, or chronic renal failure. The inclusion criteria of LNs were (1): in the regions (1-14) defined by the International Association for the Study of Lung Cancer (IASLC) guidelines (11, 12) (2); ignored SUV values on PET images.

### Model Training and Validation

**Figure 1:**
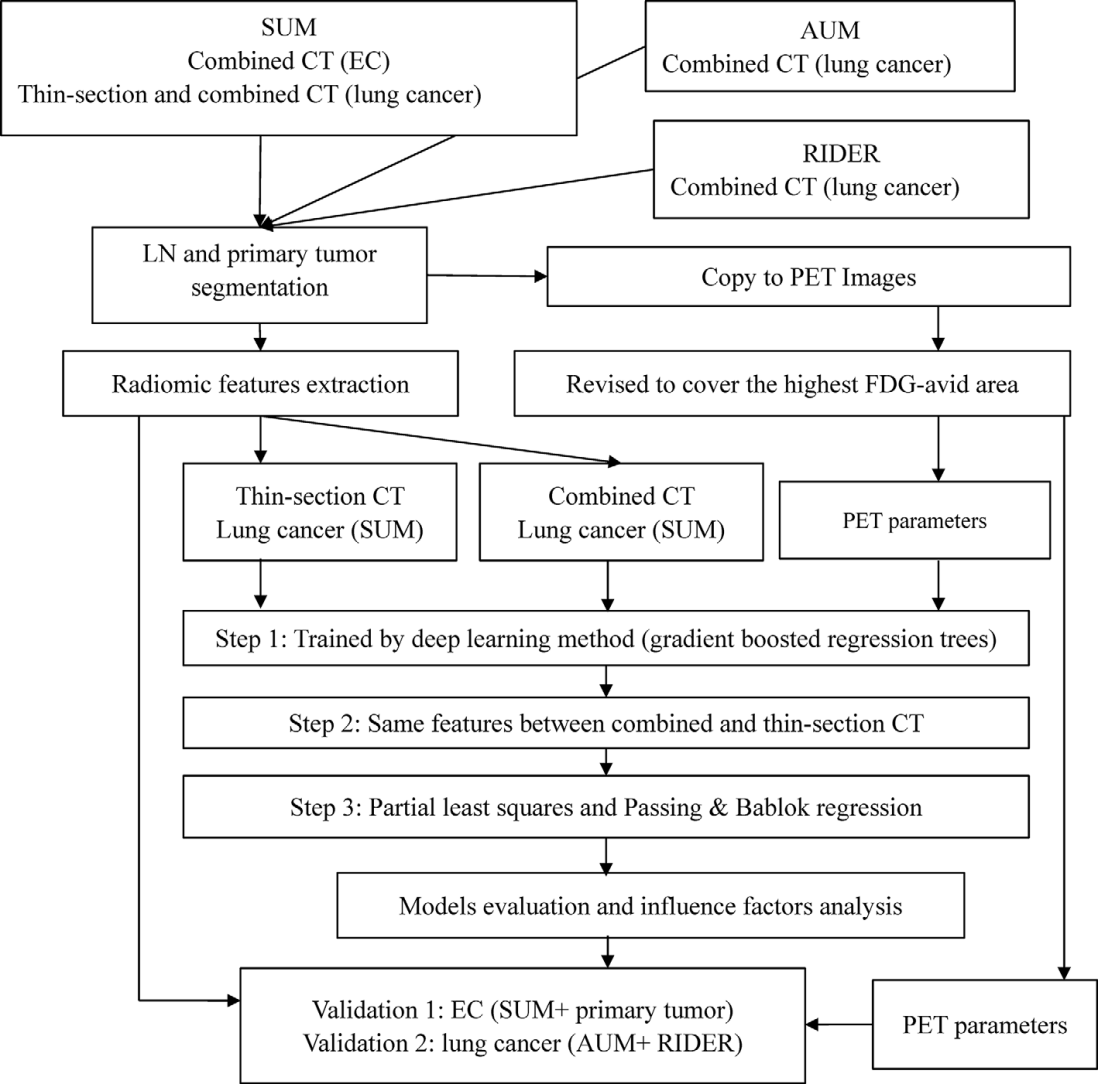
Study design and variable selection strategy.

The study design is presented in **Figure 1**. Acquisition protocols of PET and CT images (**Table S1**), CT feature extraction, model training, and model validation are included in the **Supplementary Data**.

In brief, lymph nodes and primary tumors on the combined or thin-section CT images were semi-automatically segmented. The lymph nodes (LNs) of SMU LC were the training cohort. Predictable features for estimating the SUVsum of each LN were selected and sorted by deep learning method. The candidate features were the same between the top 20 features on the combined and thin-section CT images. The estimation model was trained by the partial least squares (PLS) and the linear regression of Passing & Bablok and validated using the cohorts outlined in **Table 1**, including the primary tumor cohort. Finally, because the measurement of SUV value on PET images could be affected by some factors, such as acquisition time, injected 18F-FDG dose, tumor volume, and pathology of the primary tumor, they were collected from the metadata of images or medical records and were evaluated by the lymph nodes of SMU LC cohort.

**Table 1:** Training and validation cohorts.

	Cohort	Scanner	Validation Aim of Model
LNs of SMU LC	Training	Same	–
LNs of SMU EC	Validation	Same	Performance in LNs of other types of cancer
PTs of SMU LC and EC	Validation	Same	Performance in PTs
LNs of AMU LC	Validation	Different	Performance in LNs
LNs of RIDER LC 1	Validation	Different	Performance in LNs
LNs of RIDER LC 2	Validation	Different	Performance in LNs

LNs, lymph nodes; PTs, primary tumors; LC, lung cancer; EC, esophageal cancer; SMU, First Affiliated Hospital of Shanxi Medical University; AMU, First Hospital of Anhui Medical University; RIDER, RIDER Lung PET-CT dataset from The Cancer Imaging Archive.

### Follow-Up and Data Analysis

For each SMU cancer patient, the total SUVsum of the primary tumor and lymph nodes was calculated by trained model, and its prognostic power was evaluated and compared with the measured TLG on PET images. The patients were followed to the end of November 2019 by medical records or communications. Overall survival (OS) was defined from the day of PET/CT scan to death for any reason. The patients were staged according to the Seventh Edition of the Union for International Cancer Control American Joint Committee on Cancer System (UICC-AJCC). Age was stratified by the median age of the cohort. Treatment modalities after PET/CT scans were stratified into no treatment, chemotherapy only, radiotherapy only, and combined chemo-radiotherapy. Target drug administration was also followed, included signal transduction inhibitors, gene expression modulators, immunotherapy drugs, and others.

Right censored survival data, and model training and validation were analyzed by the R software (version 3.6.1, a language and environment for statistical computing). Besides the basic packages of the R package, others included ggplot2, Hmisc, survcomp, h2o, and et al. Two sides of P<0.05 were considered as a significant level.

## Results

### Model Training and Validation

The results of model training and validation are presented in the **Supplementary Data**. In summary, 141 features (1683 variables) were separately extracted from each lesion on the combined and/or thin-section CT images (**Figure S1** and **Table S1**). In the training cohort of lymph nodes of SMU LC (n=1239), three features (Model 1 and **Table S3**) were selected by the deep learning method, and the natural logarithm (ln) of them were regressed against the measured ln(SUVsum) by the partial least squares (PLS) regression (**Figures S2**, **S3**). After that, the linear regression of Passing & Bablok (P&B) was used to correct the estimated bias of the model. It should be noted that all the estimated coefficients of PLS and P&B regression were in relatively narrow 95% confidence intervals (95% CIs), which indicated the repeatability of the model.

In the validations of the images acquired by the same scanner (**Figure S3**), the trained model could not only be used to estimate ln(SUVsum) of the primary tumors of LC and EC patients (bias: 0.479 ± 0.557, r=0.944, P<0.001), but also that of the lymph nodes of the EC cohort (bias: 0.059 ± 0.476, r=0.896, P=0.033). Furthermore, the evaluation indices in **Figure S3** are comparable between the cohorts.

**Figure 2:**
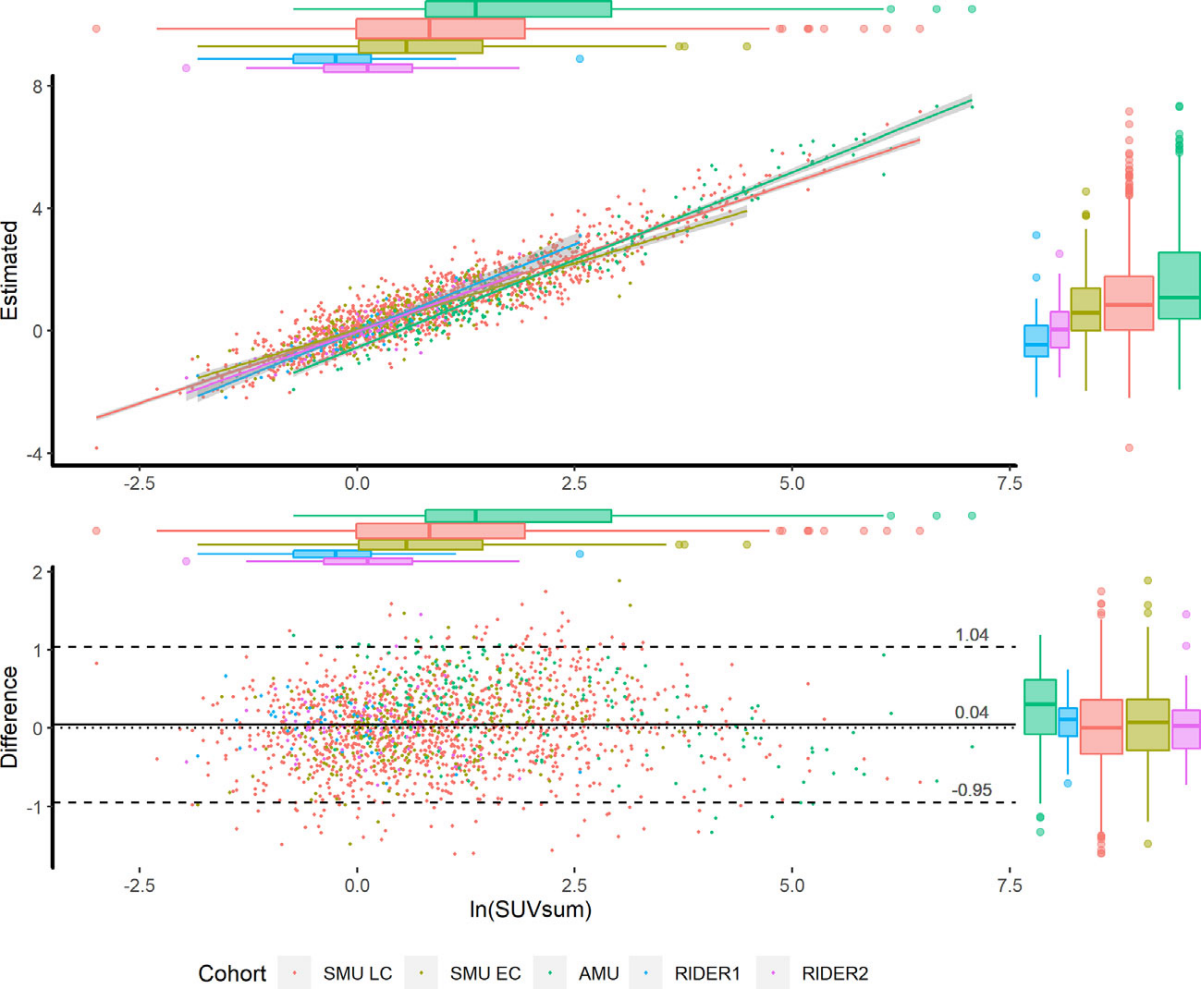
Scatter plot (upper) and Bland-Altman plot (lower) in the training and validation cohorts. According to the cohorts, the scatters are grouped by colors. The x axes of the two plots are the measured ln(SUVsum). The y axes of the scatter plot and Bland-Altman plot present the estimated ln(SUVsum) and bias, respectively. The 95% CIs of regression lines between the measurements and estimations are in grey shadow, and the scatters distribution is illustrated by the box plots on the top and right sides.

In the validations of the images acquired by different scanners, the estimation highly correlated to the corresponding measurements. However, the regression lines had slight differences that resulted from the different scanners or protocols (**Figure 2**). Therefore, for other scanners, re-estimating the coefficients of the Passing & Bablok regression could improve the performance of the model, meaning there is no need to re-perform partial least squares regression or deep learning analysis.

Additionally, the factors of acquisition time, injected dose, volume and pathology did not significantly correlate to the estimated bias of the model (**Figure S4**). Furthermore, when using SUVmax≥2.0 or 2.5 as the cutoff value of lymph node metastasis, the correlation coefficients between the measurements and the estimations were not significantly changed within an overlapped range. Therefore, the model was seldom influenced by the evaluated factors and could be used to estimate ln(SUVsum) for both benign and malignant lymph nodes.

### Patient Characteristics

Among the patients that could be followed, the median age of lung cancer (n=137) and esophageal cancer (n=56) patients were 64y (range: 38-88y) and 65.5y (range: 44-83y), respectively. Between the two cancers, the characteristics of pathology, TNM stage, and treatment modalities had significant differences (**Table 2**); therefore, the cohort was analyzed by tumor types separately.

**Table 2:** Patient characteristics.

		LC (n=137)	EC (n=56)	P
Gender	Male	97	38	0.685
	Female	18	18	
Pathology	Squamous cell carcinoma	50	51	<0.001
	Adenocarcinoma	76	5	
	Others	11	0	
Age (y)	< Median	73	25	0.276
	≥ Median	64	31	
Surgery	Yes	41	40	<0.001
	No	96	16	
Radiotherapy	Yes	47	46	<0.001
	No	90	10	
Chemotherapy	Yes	88	46	0.014
	No	49	10	
Target therapy	Yes	34	11	0.440
	No	103	45	
TNM stage	I-II	34	26	0.003
	III-IV	103	30	
mlnTLG	Median	5.1	5.2	0.547
	Range	2.3-8.4	1.9-7.3	
elnTLG	Median	5.5	5.3	0.083
	Range	2.4-10.2	2.3-8.2	

mlnTLG, Natural logarithm of measured TLG; elnTLG, Natural logarithm of estimated TLG.

To evaluate the prognostic ability of the values estimated by the model, the accumulated SUVsum of the primary tumor and lymph nodes in each patient of the SMU cohort was calculated. Between the estimated natural logarithm of TLG (elnTLG) and the measured values (mlnTLG), significant difference existed among both lung cancer (bias=0.510 ± 0.519, r=0.956, P<0.001) and esophageal cancer patients (bias=0.251± 0.463, r=0.934, P<0.001). Because the slight difference might not have a significant influence on their prognostic ability, they were analyzed as continuous variables in the following Cox regression.

After PET/CT examinations, 7 and 49 esophageal cancer patients (n=56) received surgery and combined therapy, respectively. Among the lung cancer patients, 18 did not accept any treatment, and 14, 23 and 82 individuals received surgery, chemotherapy, and combined therapy, respectively. Additionally, target drugs were administered to 34 lung cancer and 11 esophageal cancer patients. The treatment modalities had no significant difference between the pathology types of lung cancer (F=1.829, P=0.165) or esophageal cancer (F=0.137, P=0.713).

### Survival Analysis

**Figure 3:**
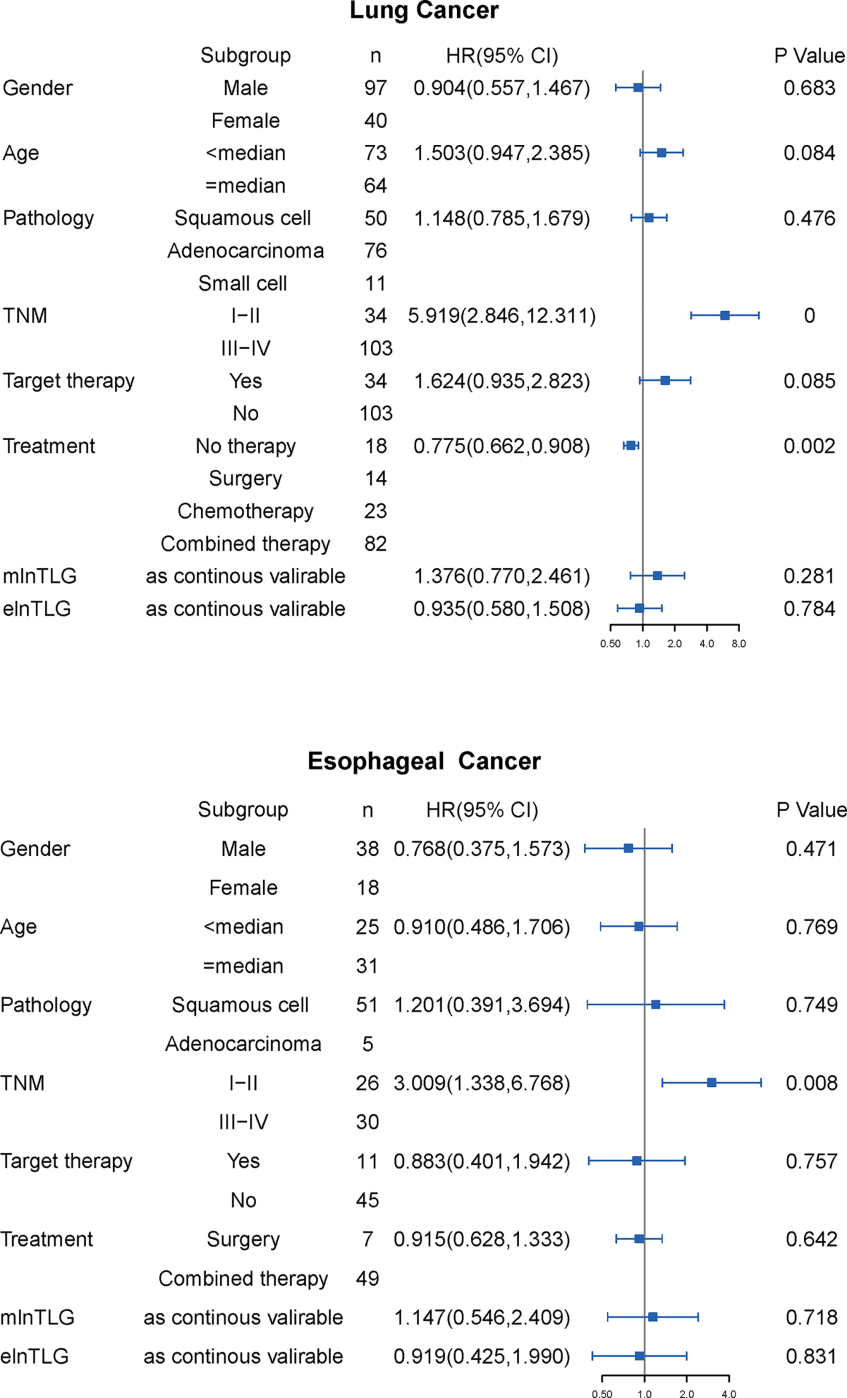
Results of univariate Cox regression analysis for lung cancer (upper) and esophageal cancer (lower). HR with 95% CI is plotted in the fourth column.

During the observation time from 30m to 106m (median: 43m), 91 lung cancer and 44 esophageal cancer patients died in the range of 0-40m (median 11 m) and 1-60m (median 15m), respectively. In the univariate Cox regression analysis (**Figure 3**), TNM stage and treatment modalities were significantly against the OS of lung cancer, and only the TNM stage was against the OS of esophageal cancer. Furthermore, the variables were also significant in the multivariate survival analysis and were used as the basic models to evaluate the prognostic ability of continuous mlnTLG and elnTLG.

**Figure 4:**
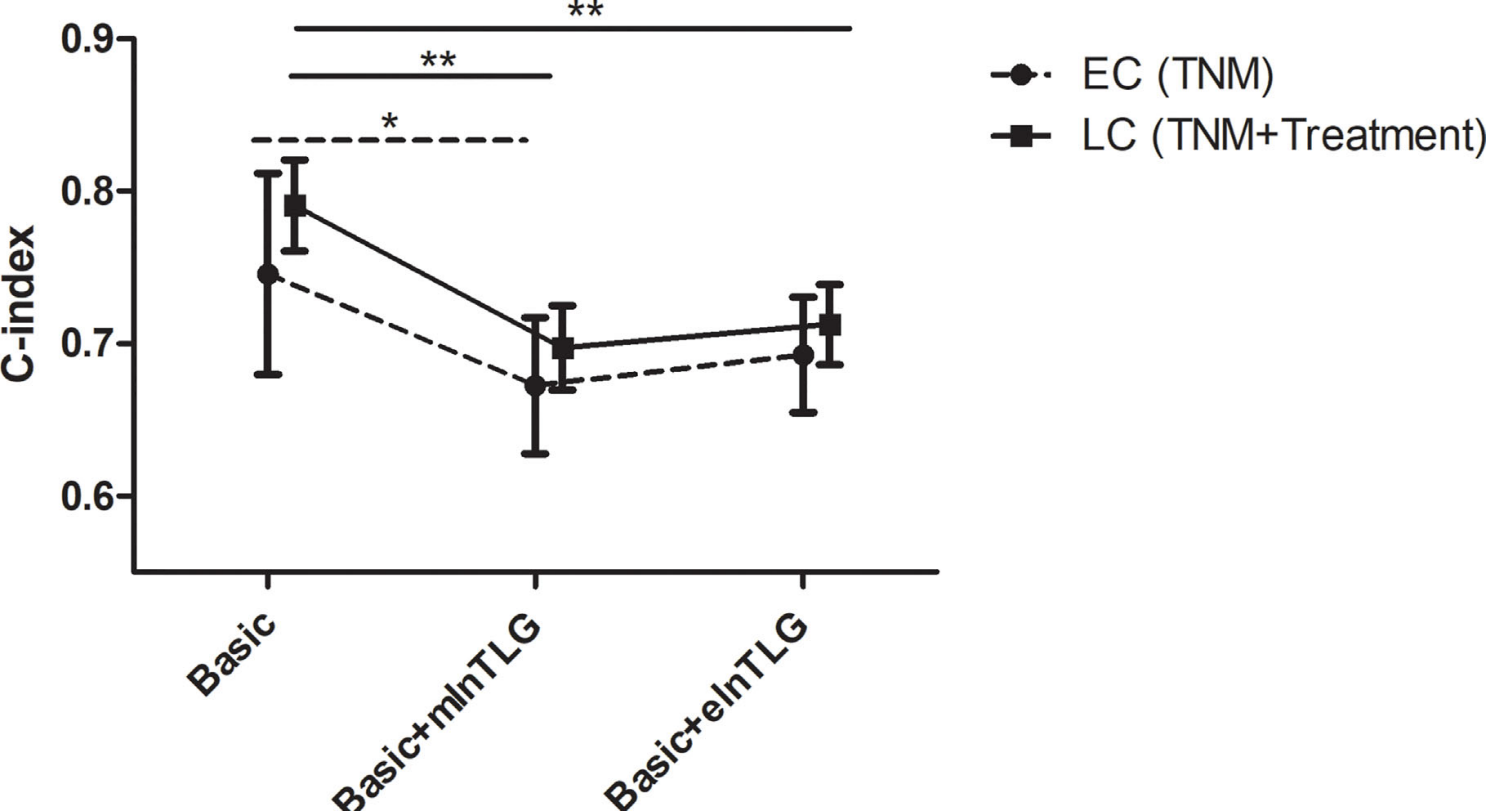
C-index in 95% CI before and after integrating mlnTLG or elnTLG. The variables of basic models for lung cancer (LC) or esophageal cancer (EC) patients are separately determined by the multivariate Cox regression (in the brackets). The significance between each two C-index is indicated by *P < 0.05 or **P < 0.01.

After integrating mlnTLG or elnTLG as continuous variables, all C-index of basic models were significantly deteriorated (**Figure 4**). For esophageal cancer, C-index did not significantly decrease by the integration of elnTLG (P=0.109), which resulted from the limited cases. Furthermore, the C-index of the models containing elnTLG was slightly higher than those containing mlnTLG, but without significance.

**Figure 5:**
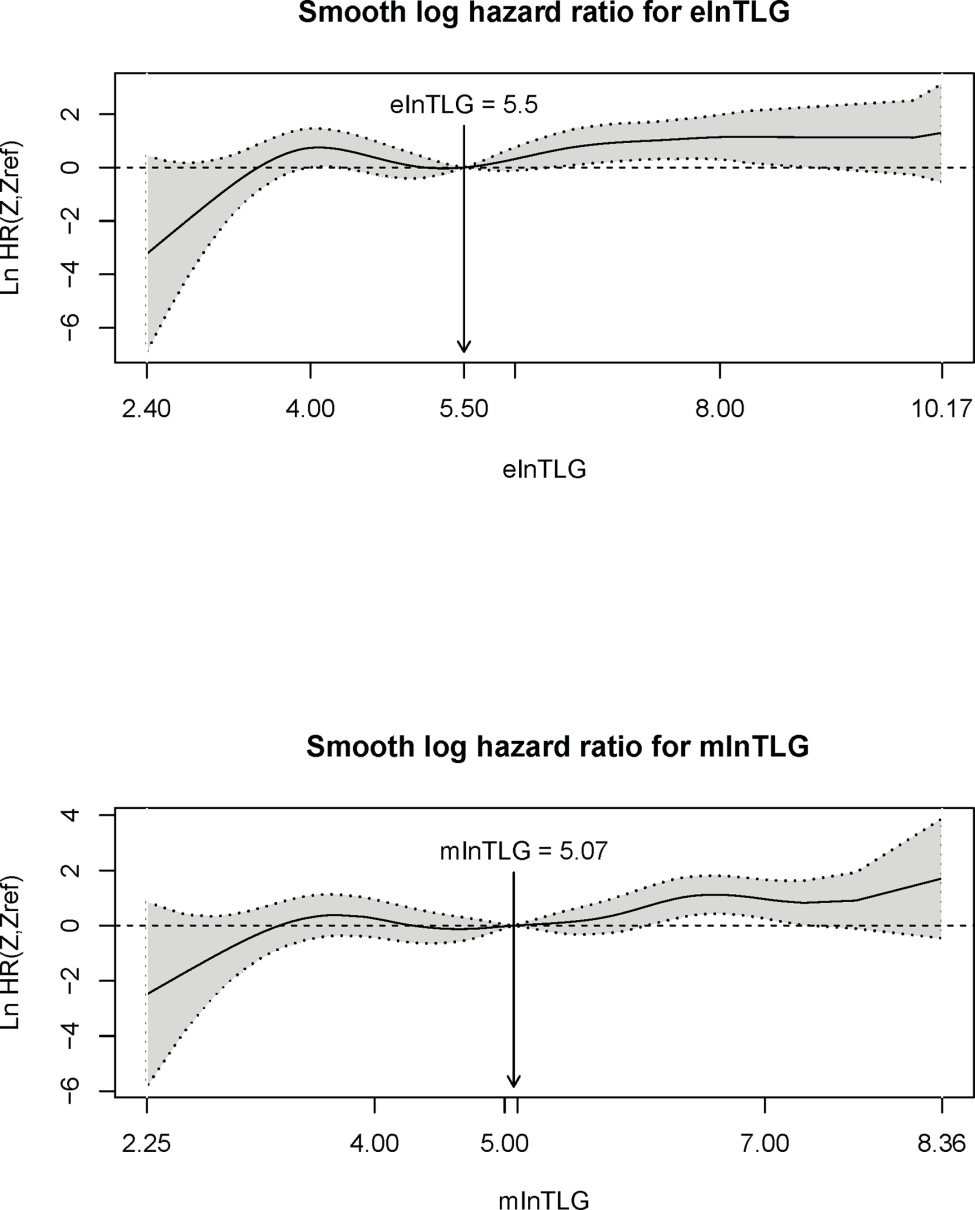
Plots of continuous elnTLG (upper) and mlnTLG (lower) against ln(HR) in lung cancer patients. The vertical lines and arrows indicate the median reference value of hazard ratio curves which are defined by the Akaike information criterion (AIC) values (756.11 *vs*. 756.16).

According to the method from Liu et al. (13), the correlation between the continuous variables (ie. elnTLG and mlnTLG) and OS were analyzed and compared among the lung cancer patients. **Figure 5** indicates that the two variables are non-linearly associated with the logarithm of HR (lnHR). In general, lnHR increased with the increase of either elnTLG or mlnTLG, and the overall shapes of the curves were quite alike. Additionally, the median reference values of hazard ratio curves were closed to each other (5.50 vs. 5.07). Similar results were also found in the esophageal cancer patients, except that both elnTLG and mlnTLG were negatively correlated to lnHR (**Figure S6**, **Supplementary Data**). Therefore, in evaluating the prognosis of lung cancer patients after PET/CT examinations, the estimated TLG from CT images was similar to those measured on PET images.

## Discussion

In this study, among the lung cancer and esophageal cancer patients, the CT radiomics model trained from lymph nodes could be used to calculate the natural logarithm of SUVsum of primary tumors and lymph nodes. Furthermore, in predicting the outcome of lung cancer patients, total lesion glycolysis (TLG) calculated by the model was similar to those measured on PET images. It should be noted that, for different scanners and protocols, a simple linear regression rather than deep learning analysis could be used to correct the bias. Additionally, the model was seldom affected by the evaluated factors of injected dose, acquisition time, LN volume, pathology, and even lymph nodes metastasis or not. Above all, our results illustrated the possibility of estimating quantitative values by radiomic models.

With the development of computer science and technology, hundreds of radiomics features can be extracted from medical images. A previous study from Giesel et al. (14) indicated that, whether lymph nodes metastasized or not, the density of lymph nodes on the CT images was associated with FDG uptake. Furthermore, features of uniformity and entropy with coarse filtration negatively correlated to SUVmean (r=-0.754 and -0.748) (15). These results are consistent with ours, and the three features of our model were calculated from mass, volume, surface area, and the number of voxels (**Supplementary Data, Table S2**), those related to LN’s density. Therefore, as indicated by our results, the trained and validated model could be used to estimate the SUVsum of each lymph node or primary tumor from CT images alone.

Recently, TLG was proposed as a better prognostic index than SUVmax among most cancer patients (7, 16, 17). Therefore, to illustrate the performance of our model in predicting patient outcomes, the lung cancer, and esophageal cancer patients were followed. Our results indicated that there was no significant difference between the estimated and the measured TLG; however, the estimated values even performed slightly better than the measurement. Although the TLG of this study included some benign lymph nodes, their faint FDG uptake could not significantly affect the accumulated values which included those from metastases foci and primary tumor. Therefore, the estimation method could be a complementary and valuable feature for CT scans that would be beneficial for patients by avoiding unnecessary radiation exposure and reducing medical expenditure.

In molecular imaging-guided radiation therapy (MIGRT), the delineation of gross target volumes (GTV) on CT is improved by molecular imaging technology, such as PET and SPECT images. The simple way is to manually delineate CT GTV and revise its boundary by visual interpretation of PET (18). However, the method suffers from intra- and inter-observer variability. Therefore, in research, metabolic tumor volume is usually defined by a fixed threshold of SUVmax (19). Because the purpose of this study was to estimate SUVsum within CT VOI, the threshold method was substituted with directly copying CT VOI to PET images. The delineation method might help in estimating the SUV of each pixel based on CT image alone and mimicking the uptake of FDG.

To train a repeatable model, as pre-described in the **Supplementary Data**, only the duplicated features between the combined and thin-section CT serials were adopted as candidates, and further selected by the partial least square regression method. The model could even be used for the estimation of the primary tumor and lymph node of the esophageal cancer cohort recruited from the same medical center, and also performed well in all validation cohorts.

The advantage of the logarithmic estimation was that it was seldom affected by the evaluated factors, did and did not include lymph node metastasis. Because pathological evidence for LNs was not easy to prove, we alternately used the SUVmax=2.0 (or 2.5) as the threshold of benign and malignant ones. The correlation coefficients between the measured and estimated values were not changed according to the stratifications. Additionally, no different serial scatters appeared on the Bland-Altman and scatter plots of Model 1 (**Figure 2**). Therefore, whether LNs metastasis or not, the natural logarithm of SUVsum could be estimated by the single model.

Additionally, our data suggested that it was possible to estimate the content of lesions by a radiomics model *in vivo*. Although the method needed to preset radiomics models for different equipment, it was easily performed by researchers or even equipment vendors through a simple linear regression. The advantage of the method was that the changes of the content could be repeatedly observed *in vivo*, and would widen the application range of radiomics.

In summary, our study indicated that several CT radiomic features can be used to estimate the logarithm of SUVsum acquired by the same scanner, and the coefficients of the models could be corrected by a simple linear regression for different scanners or protocols.

## Conclusion

Total lesion glycolysis can be estimated by three CT features with particular coefficients for different scanners, and it similar to the measured values in predicting the outcome of cancer patients.

## Data Availability Statement

The raw data supporting the conclusions of this article will be made available by the authors, without undue reservation.

## Ethics Statement

The studies involving human participants were reviewed and approved by First Affiliated Hospital of Shanxi Medical University and the First Affiliated Hospital of Anhui Medical University. Written informed consent for participation was not required for this study in accordance with the national legislation and the institutional requirements.

## Author Contributions

Conceptualization, SL. Methodology, HS and XH. Software, LZ. Validation, XX and JC. Investigation, PW and LL. Resources, ZW. Data curation, CZ. Writing—original draft preparation, HS. Writing—review and editing, SL. Supervision, S.L. Project administration, S.L. Funding acquisition, H.S. All authors contributed to the article and approved the submitted version.

## Funding

The work was funded by grants from Collaborative Innovation Center for Molecular Imaging, Precise D&T Center (Grant No. MP201604) and Research Project of Health Commission of Anhui Province (Grant NO. AHWJ2021b148). Funding organizations had no role in the design, implementation, interpretation, and publication of the study.

## Conflict of Interest

The authors declare that the research was conducted in the absence of any commercial or financial relationships that could be construed as a potential conflict of interest.
