# Supplementary material for: Total Lesion Glycolysis Estimated by a Radiomics Model From CT Image Alone
Source: Front Oncol. 2021 Jun 17;11:664346. doi: 10.3389/fonc.2021.664346 (PMC8247448; doi:10.3389/fonc.2021.664346)
Supplement: Supplementary file 1 [file DataSheet_1.doc]

Supplementary Data

**Materials and Methods**

**Patient Preparation and Acquisition Protocols**

After fasting for at least six hours and measuring blood glucose levels (<150mg/ml), the enrolled patients were intravenously injected ^18^F-FDG (3.7-5.5MBq/kg). After resting for about 50-70 min, PET/CT images of SMU and AMU were acquired by the GE Discovery VCT scanner (GE Healthcare, WI, USA) and the True Point 64 Biograph PET/CT scanner (Siemens Healthineers, Erlangen Germany), respectively. The acquisition protocols of the two centers are similar, and are listed in Table S1. In summary, combined CT and PET images were subsequently acquired. PET images were reconstructed using CT attenuation maps, which were bi-linearly transformed according to the scaling factors for materials with Hounsfield units. The standardized uptake value (SUV) of pixels was normalized to the patient’s body weight. At the 1^st^ Affiliated Hospital of SMU, an additional thin-section CT series was acquired for lung cancer patients.

**Table S1. PET and CT acquisition protocol**

|  |  | SMU | | AMU |
| --- | --- | --- | --- | --- |
|  |  | Combined | Thin-section | Combined |
| CT | Tube voltage (kV) | 120 | 120 | 120 |
|  | Tube current (mA) | 60 | 250 | 56-150 |
|  | Pitch | 0.989 | 0.989 | 0.966 |
|  | Slice thickness (mm) | 3.75 | 1.25 | 5 |
|  | Spacing (mm) | 2.75 | 1.25 | 2.75 |
|  | Rotation time (s) | 0.8 | 0.8 | 0.88 |
|  | Convolution kernel | Chest | Chest | B10f-20f |
|  | Matrix | 512×512 | 512×512 | 512×512 |
| PET | Scan speed (min/bed) | 2 | NA | 5.8 |
|  | Reconstruction | OSEM | NA | OSEM |
|  | Slice thickness (mm) | 3.75 | NA | 2 |
|  | Subsets | 20 | NA | 20 |
|  | Iterations | 2 | NA | 4 |
|  | FWHM (mm) | 6.00 | NA | 5.00 |
|  | Matrix | 256×256 | NA | 256×256 |

NA: Not Applicable

**Image Segmentation and Features Extraction**

All images were in DICOM format, and were transported into a personal computer. At first, LNs on cross-sectional CT images (short-axis diameter ≥ 3mm) identified by two experienced radiologists were grouped according to the International Association for the Study of Lung Cancer (IASLC) guidelines . Subsequently, using the 3D-Slicer (version 4.8), a volume of interest (VOI) for each LN was segmented semi-automatically (Figure S1) . Finally, the IBEX software (version 2.0) was used to extract CT features that included the first-order, spatial autocorrelation, second-order, high-order, absolute gradient, shape and size . Additionally, for the SMU LC and EC patients, the features of visible primary tumors were extracted to validate the model of LNs. Although our images were from several medical centers, only the original features were used in the following analysis.

Figure S1: Image segmentation (upper row) and SUV measurement (lower row).


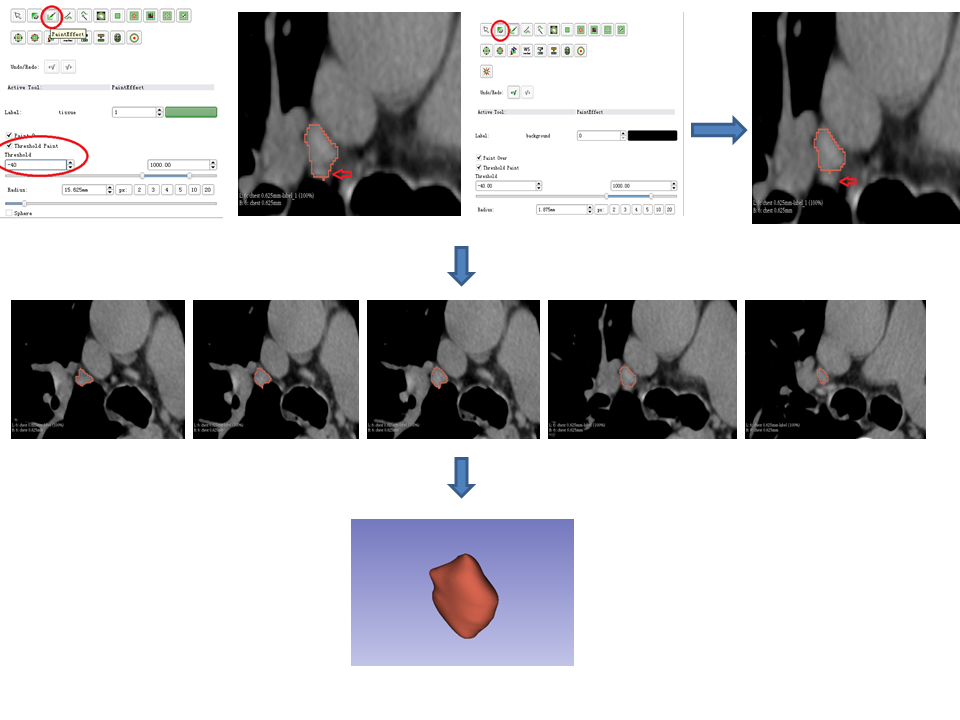


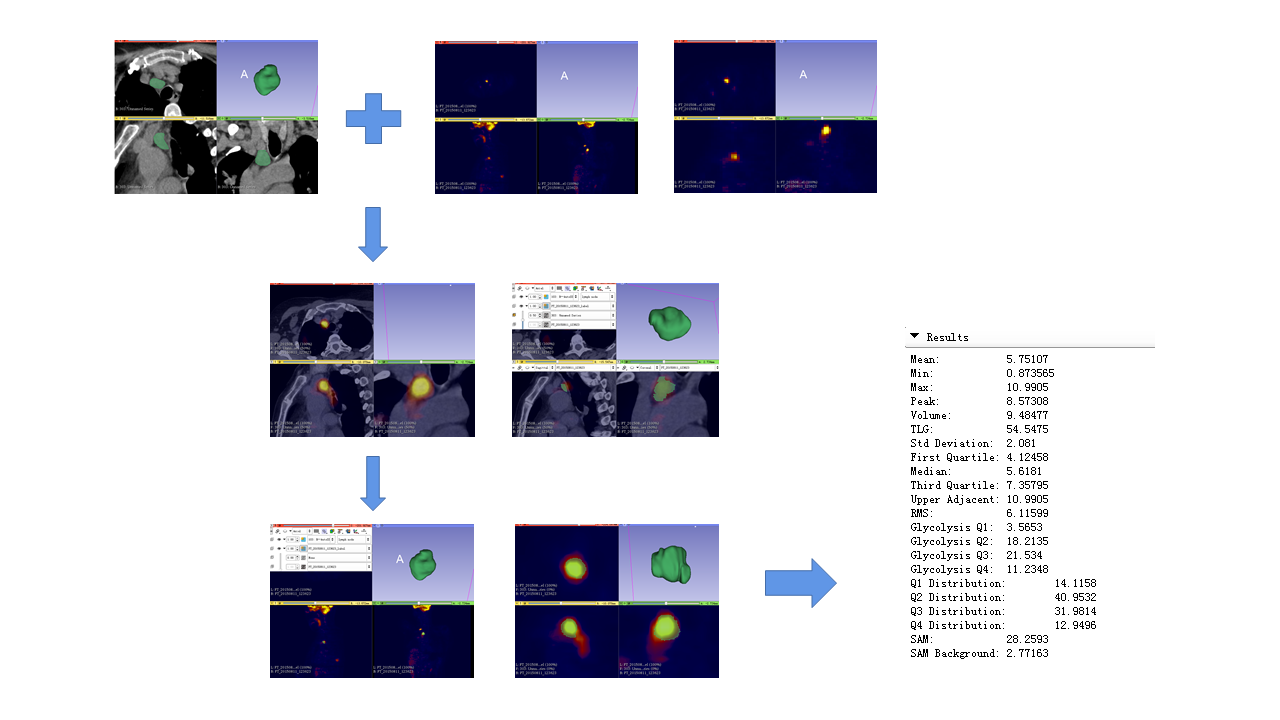


To measure the corresponding SUVsum of lymph nodes and primary tumors, VOI on the combined CT was copied to PET series (Figure S1). It was adjusted to better cover the highest FDG-avid area when presenting an obvious fusion error. After that, SUVsum of lymph nodes and primary tumors were measured and the natural logarithm of SUVsum was calculated, i.e. ln(SUVsum).

**Variable Selection**

Study design and variable selection strategy are presented on Figure 1. In the training cohort (LNs of SMU LC), CT features did not linearly correlate to the measured SUVsum but did so after a natural logarithm transformation. Therefore, the measured ln(SUVsum) was treated as dependent variables, and the logarithmically transformed CT features were independent variables. The combined and thin-section CT features were separately analyzed by the gradient boosted regression trees (GBRT) of the deep learning method, and the top 20 variables were sorted by the parameter of variable importance (VIMP), which was a comparable measurement of the contribution of a variable to the regression. The variables were selected by the 10-fold cross-validation, and other parameters were number of trees=10, fewest allowed observations in a leaf=2, maximum tree depth=3, learn rate=0.2, and distribution=gaussian. The candidate features were identified as the duplicates among the top 20 ones extracted from the two types of CT serials. After that, the partial least squares (PLS) regression which was internally validated by the leave one out method was used to estimate the coefficients of the model. Considering the multicollinearity of the features, the regression were also used to furtherly select variables from the candidates indicated by score and adjusted R square (R^2^) .

**Model validation**

In this study, the desired model made up of several reliable CT features, and variable coefficients could be adjusted according to scanners or protocols. Therefore, besides the training cohort (SMU LC), the external validation sets included LNs of SMU EC, primary tumors of SMU LC and SMU EC, and LNs of AMU LC and the RIDER Lung PET-CT datasets (Table 1). Both the SMU EC and SMU LC images were acquired with the same scanner and protocol; therefore, the related cohorts would validate the model for a given scanner (validation 1). Furthermore, other cohorts could evaluate whether the model could be used for other scanners (validation 2).

In the validations, besides the Bland-Altman plots, the indices of accuracy (bias or mean) and precision (SD, correlation coefficient, and RMSE) were calculated and compared. After that, some influence factors of the model, including LN metastasis, FDG injected dose, acquisition time after injection, pathology classification, and LN volume were analyzed by the correlation matrix method and scatter plots. Data were analyzed by the open source software of the R package (version 3.5.1). A two-sided p-value less than 0.05 was considered as a significant difference.

**Results**

**Patient Characteristics**

Patient characteristics of SMU, AMU, and the RIDER Lung PET-CT dataset are listed in Table S2. Among the SMU patients, 256 primary tumors were visible on PET/CT images (LC n=200 and EC n=56). Between the LC patients from SMU and AMU, most characteristics were comparable, except the injected dose (t=7.607, P<0.001) and number of LN (t=2.649, P=0.010). However, except for gender (*x*^2^=5.300, P=0.151), acquisition time (t=0.523, P=0.667) and body height (t=1.841, P=0.140), other characteristics of RIDER cohorts were significantly different from other cohorts. Additionally, the SMU EC patients are inclined to have older age, lighter weight, smaller volumes of LNs, and lower values of SUVsum. Although significant differences existed among the cohorts, they could be explained by the different medical centers or diseases.

Table S2: Patient characteristics

|  |  | SMU | SMU | AMU | RIDER |
| --- | --- | --- | --- | --- | --- |
|  |  | LC (n=206) | EC (n=61) | LC (n=36) | LC (n=45) |
| Gender (M/F) |  | 143/63 | 39/22 | 28/8 | 25/20 |
| Age (y) | Mean±SD | 63.3±9.7 | 66.1±9.7 | 63.1±15.7 | 68.7±9.7 |
| Body height (cm) | Mean±SD | 167.2±7.2 | 165.8±8.3 | 169.5±4.9 | 169.3±11.1 |
| Body weight (kg) | Mean±SD | 65.7±11.1 | 59.8±9.1 | 63.6±12.2 | 77.1±18.1 |
| Dose (MBq/kg) | Mean±SD | 5.0±1.0 | 5.1±0.8 | 4.3±0.3 | 6.8±1.8 |
| Acquisition | Mean±SD | 69.8±23.9 | 69.4±25.1 | 71.0±32.8 | 65.1±17.4 |
| time (min) | Median | 65.5 | 65.0 | 65.0 | 60.0 |
|  | Range | 32-202 | 31-155 | 33-108 | 43-128 |
| Number of LN | n(Range) | 1239 (1-17) | 301 (1-16) | 168 (1-13) | 242 (2-13) |
|  | Median | 5 | 4 | 4 | 4 |
| Volume (ml) | Mean±SD | 2.4±4.5 | 1.5±1.4 | 2.9±3.5 | 0.9±0.6 |
|  | Median | 1.3 | 1.1 | 1.7 | 0.7 |
|  | Range | 0.7-84.1 | 0.2-13.8 | 0.3-20.9 | 0.16-5.7 |
| SUVsum | Mean±SD | 8.2±27.7 | 4.2±7.5 | 8.8±15.6 | 1.6±2.7 |
|  | Median | 2.3 | 1.8 | 3.3 | 1.1 |
|  | Range | 0.05-644.0 | 0.2-88.4 | 0.5-103.4 | 0.1-36.2 |

S/A: Squamous cancer/ Adenocarcinoma

**Variable Selection and Model Training**

From each of LN or primary tumor on the combined or thin-section CT images, 141 features (1683 variables) were extracted. In the training cohort (LNs of SMU LC patients, n=1239), CT features did not linearly correlate to the measured SUVsum, but did so after natural logarithm transformation. Using the gradient boosted regression trees (GBRT) of the deep learning method, the radiomics features from the combined or thin-section CT images were separately regressed against ln(SUVsum) of LNs, and the top 20 features were sorted by the parameter of variable importance (VIMP) which was a comparable measurement of variable contributed to the models. Between the two CT serials (Figure S2), six features were duplicated, and were as the candidates for the estimation.

Figure S2. Histogram of the gradient boosted regression trees (GBRT) regression for the combined CT (upper) and the thin-section CT (lower). The top 20 variables are list along the y axis, and are scored by the parameter of variable importance (VIMP). The VIMP in the range of 0-1 is scaled along the x axis as the comparable indicator of contribution to the regression.


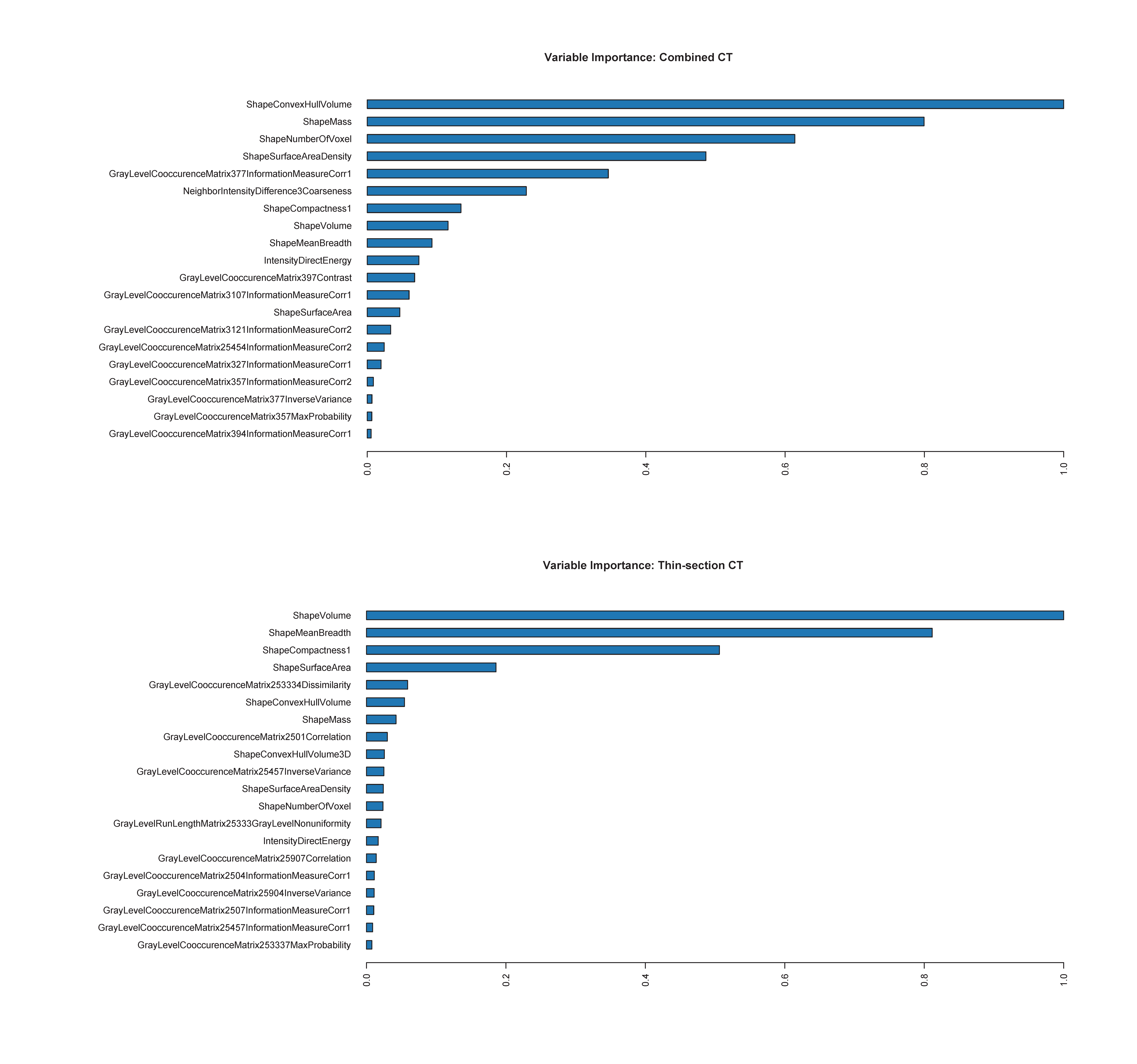


Considering the multicollinearity of the candidate features against ln(SUVsum), the partial least squares (PLS) regression was used to further select variables and to estimate coefficients of the model. Figure S3A and S3B indicate that, after integrating the 3^rd^ variable, the adjusted R^2^ and scores cannot be obviously improved; therefore, the 3 features in Model 1 are used for the estimation.


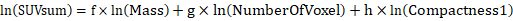


Model 1

where f, g and h are partial least squares regression coefficients, and the computation of the features are listed in Table S3.

Table S3. Explanation of radiomic features in Model 1

| Feature | Calculation |
| --- | --- |
| Mass | Calculated by CT Hounsfield unit |
| Number of voxel | Number of voxels (edge voxel is considered as EdgeVoxelFraction×Voxel) |
| Compactness1 | Volume/(sqrt(pixels)×(SurfaceArea) ^2/3^) |

Figure S3: Coefficients estimation and validation of Model 1. In further variable selection of the partial least squares (PLS) regression, the plots of A and B are curves of adjusted R^2^ and scores, respectively. The plots of C and D are coefficients estimated by the PLS and Passing & Bablok (P&B) regression, respectively. The validation results of cohorts are present on E.


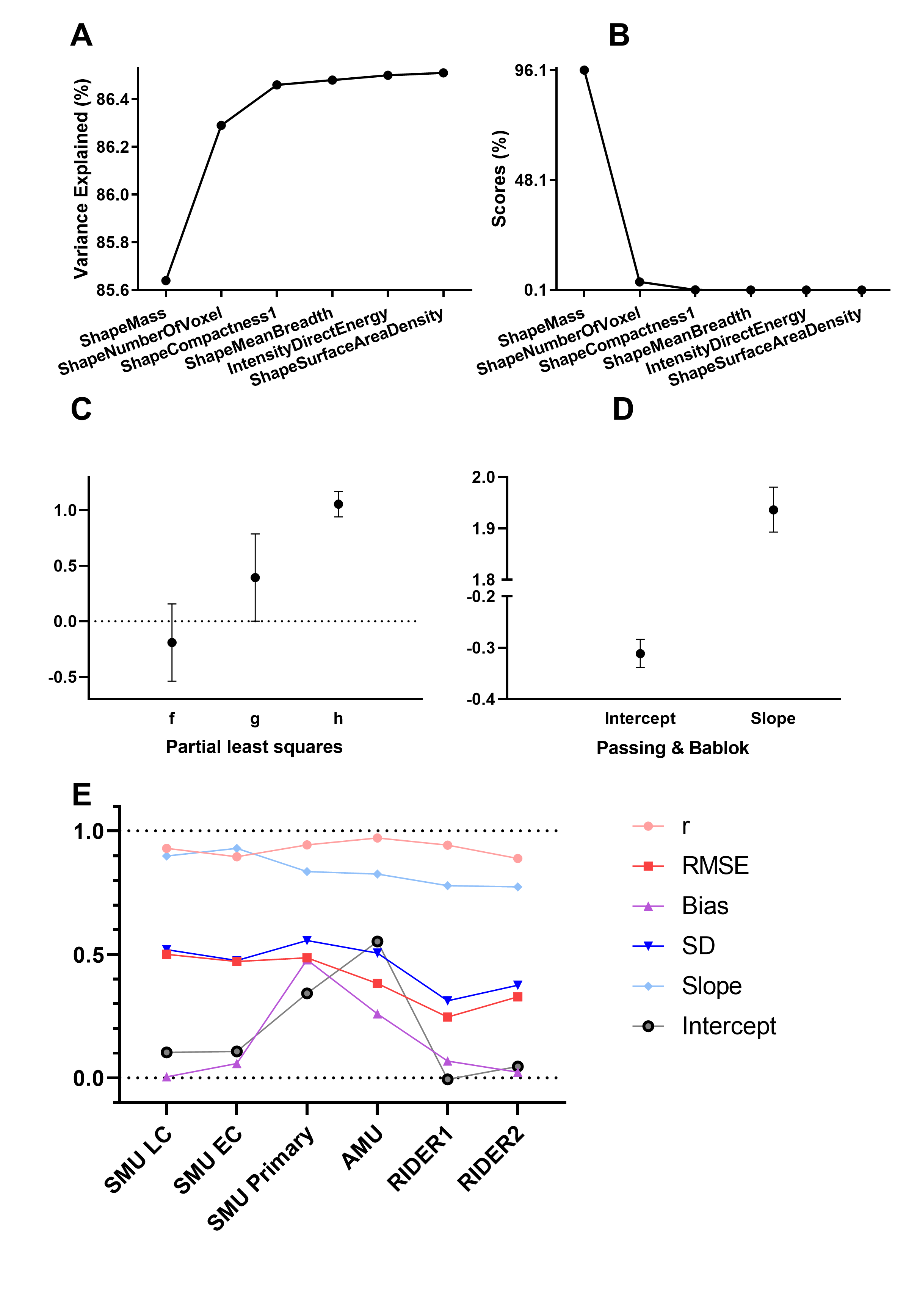


The coefficients of Model 1 was estimated by the PLS method which was internally validated by the leave one out (LOO) technique (Figure S3C). In the regression, a constant was not included for worsening the estimation of other coefficients; therefore, the linear regression of Passing & Bablok regression (P&B) was used to correct the omitted constant (Figure S3D). It should be noted that, in both the PLS and the P&B regressions, all coefficients were in relatively narrow 95% confidence intervals (95% CI) which indicated the repeatability of the models.

**Model Validation**

In the validation 1, Figure S3E indicates that the estimated and the measured ln(SUVsum) of training cohort do not have significant differences and highly correlate to each other (bias=0.004±0.520, r=0.929, P=0.873). Furthermore, the model could accurately estimate the values of lymph nodes of EC patients (bias= 0.059±0.476, r=0.896, P=0.033), and that of primary tumors of EC and LC patients (bias=-0.479±0.557, r=0.944, P<0.001). Additionally, other indices of the validation 1 were comparable among the cohorts, and the intercepts and slopes of the regression line between the estimated and measured values were close to 0 and 1, respectively. This meant that the estimations were much closer to the measurements.

In the validation 2, the cohorts from other medical centers confirmed the reliability of the estimation model. Figure 2 indicates that the estimated and measured values are linearly correlated, and there are slight differences among the intercepts and slopes of the regression lines between them. Therefore, for other medical centers, the performance of the trained model could be improved by a linear regression of Passing & Bablok without the need to re-estimated the coefficients by PLS.

**Influence factors**

Using the cross-correlation matrix, the absolute bias (absolute difference between estimations and measurements) of the training cohort was plotted against influence factors included the injected dose, acquisition time after the injection, LN volume, and pathology classification of primary tumors. Figure S4 indicates that the model seldom affects by the factors.

Figure S4: Cross-correlation matrix of influence factors against absolute bias. The matrix plots are stratified by the pathology of primary tumors and indicated by the colors of pink (squamous cell carcinoma, SCC), green (adenocarcinoma) and blue (others). The x and y axes of each panel are instructed by the labels on the top and the right sides, respectively. The correlation coefficients for every two factors are also printed, and the black (Cor) indicates that for all patients.


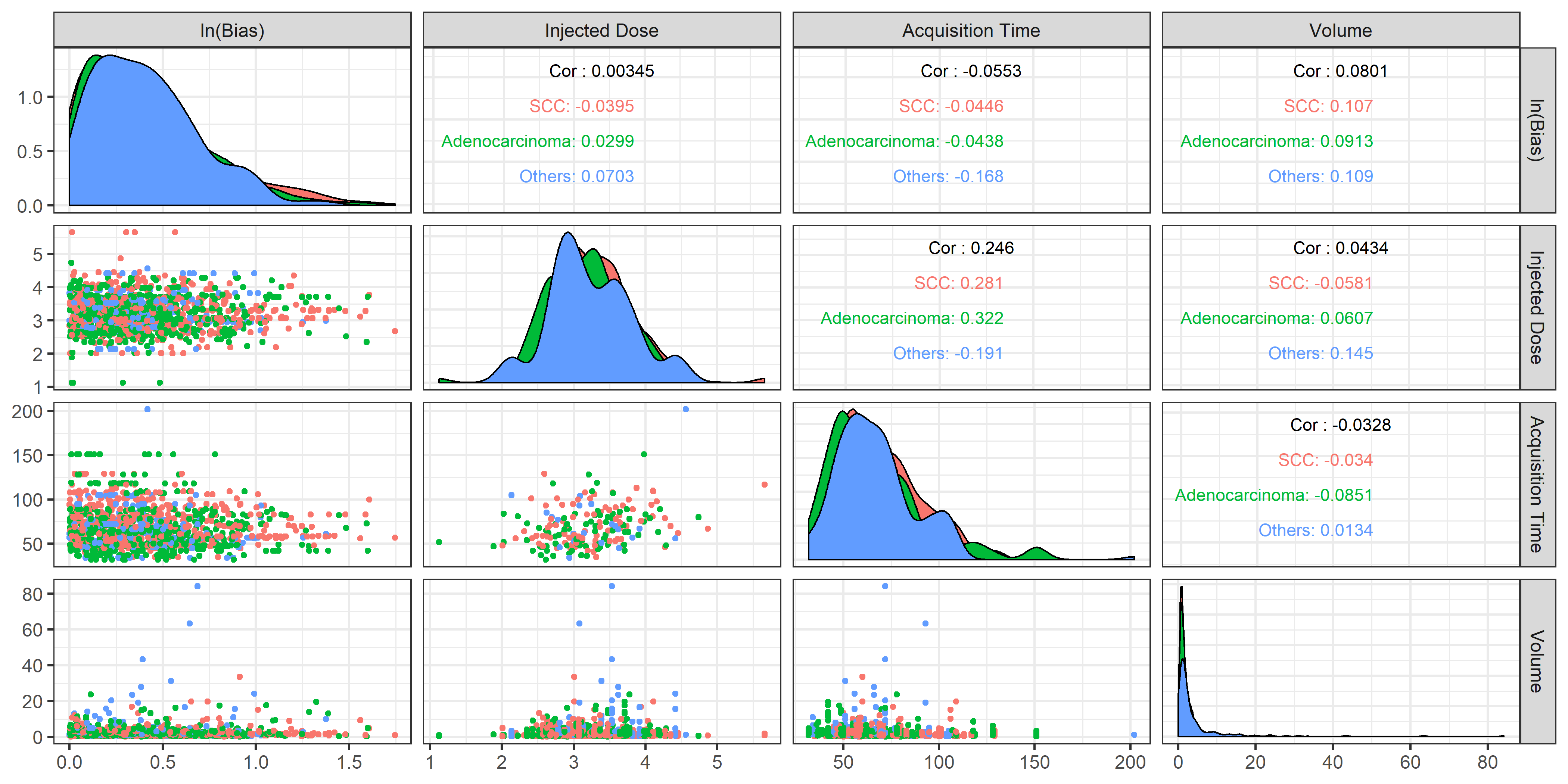


Among the recruited cancer patients, all segmented LNs were not definitely metastasized, especially for those with faint FDG uptake; therefore, it was necessary to explore its influence on the trained model. On Figure S5 of the Bland- Altman (left column) and scatter plots (right column), the scatters are grouped by the thresholds of SUVmax≥2.0 or 2.5 which is as the cutoff value of LN metastases, and indicate that the estimations linearly correlated to the measurements within a narrow range. Between the estimated and the measured values, the correlation coefficients for SUVmax≤2.0 and >2.0 LNs were 0.910 (n=471, 95%CI: 0.892- 0.926) and 0.935 (n=769, 95%CI: 0. 924- 0.945), and those for SUVmax≤2.5 and >2.5 were 0.913 (n=616, 95%CI: 0.897- 0.927) and 0.934 (n=624, 95%CI: 0.922- 0.945), respectively. Therefore, neither the threshold of SUVmax=2.0 nor SUVmax=2.5 could significantly affect the correlation coefficients, and their 95% CIs were overlapped more or less. Additionally, Figure S5 (lower row) also indicates that the model trained from LNs could also be used to estimate ln(SUVsum) of primary tumors.

Figure S5: Bland-Altman plots (left column) and scatter plots (right column) of the training cohort. Scatters are grouped by different colors. The upper and median rows are for the SUVmax threshold =2 and 2.5, respectively. The lower row is the plots for the comparison of primary tumor and lymph nodes. All the x axes of the two kinds of plots are the measurement. The y axes of Bland-Altman and those of scatter plots present bias (difference between measurements and estimations) and estimations, respectively.

**

**

Figure S6: Plots of continuous elnTLG (upper) and mlnTLG (lower) against ln(HR) in the esophageal cancer patients. The vertical lines and arrows indicate the median references value of hazard ratio curves which are defined by the Akaike information criterion values (299.29 vs. 300.95).


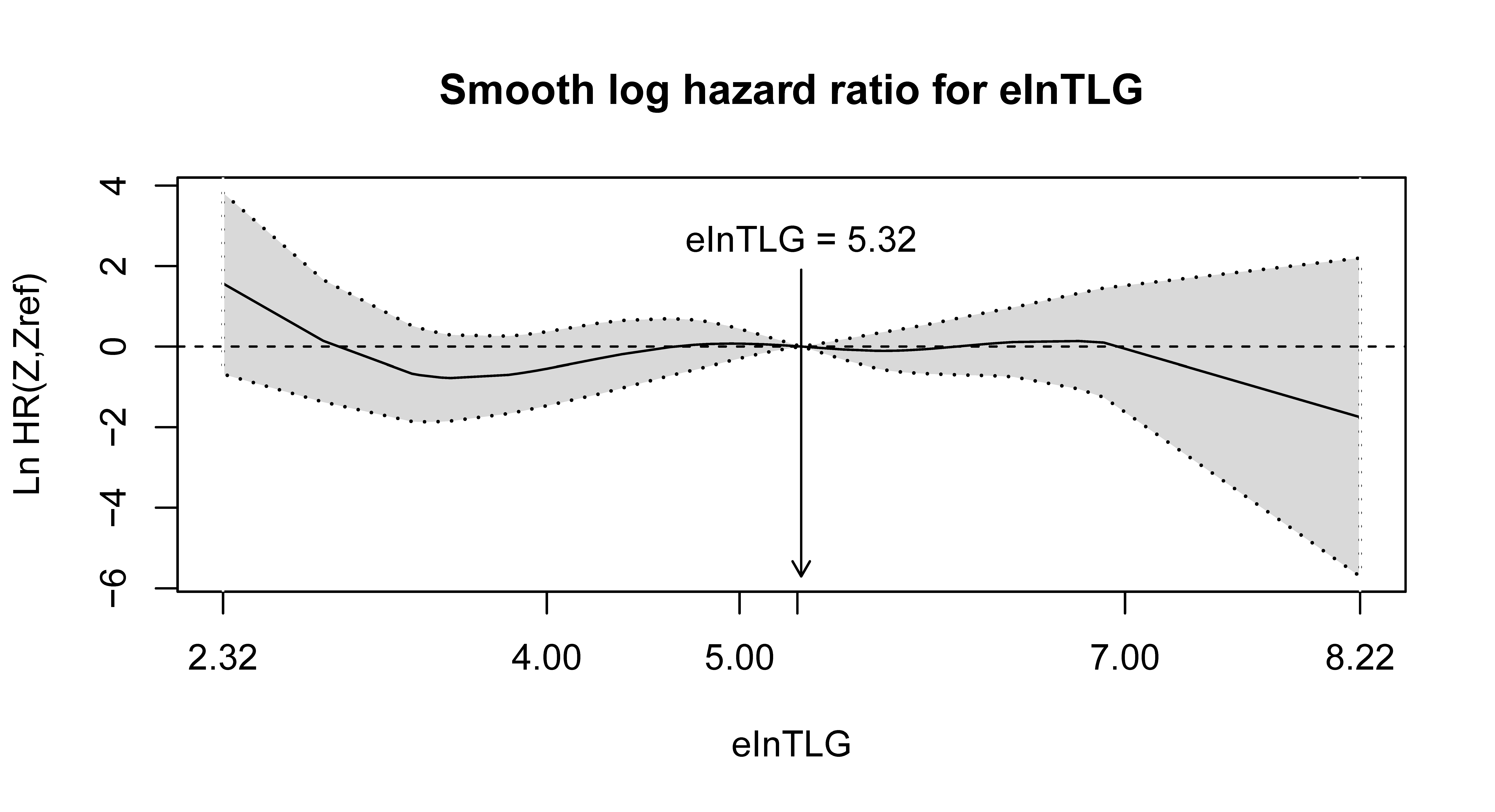


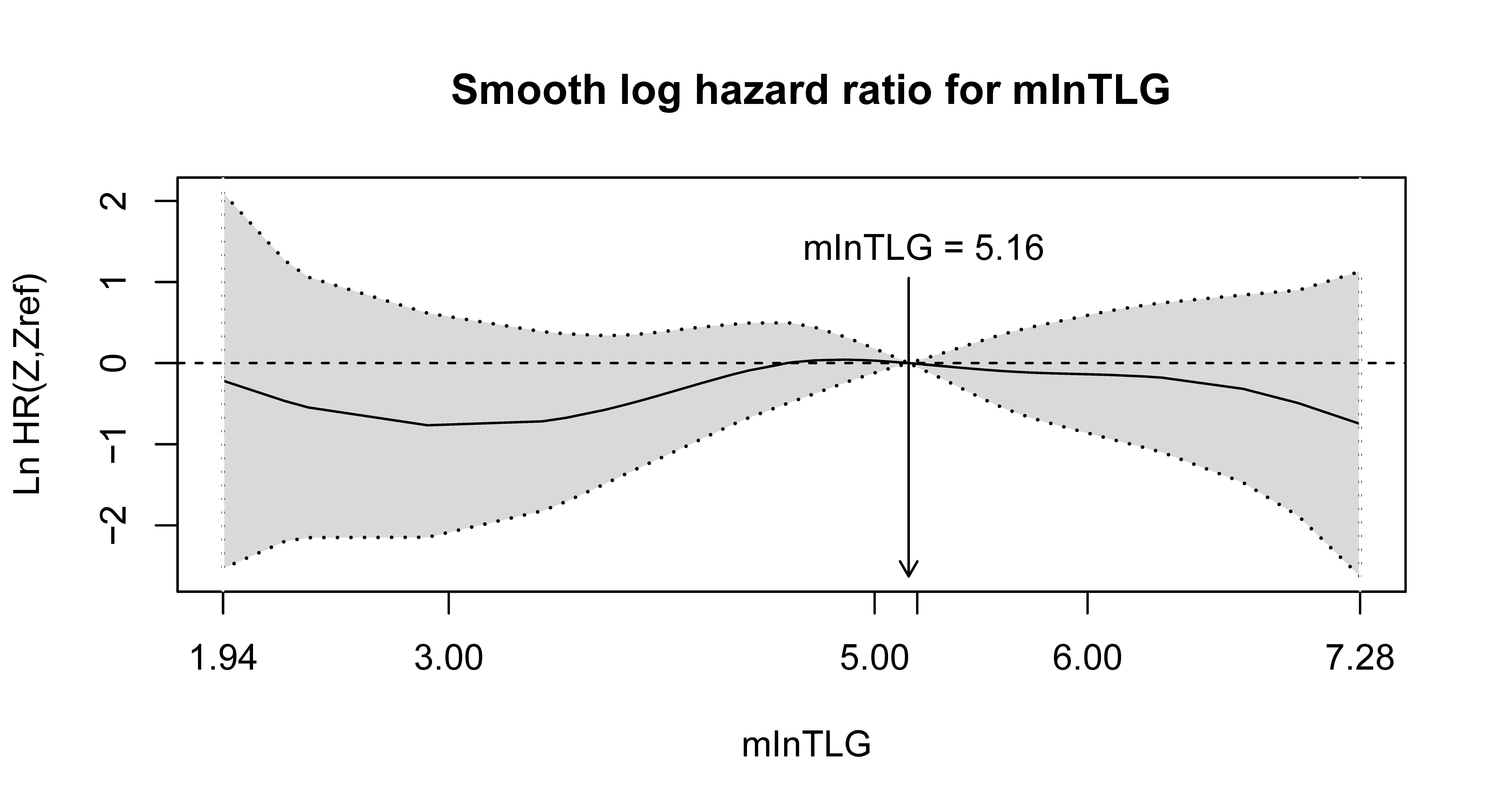


**Reference**

**1.** Xia Y, Ma Y, Arias S, Lee H, Wang KP. Utilization of the International Association for the Study of Lung Cancer and Wang's nodal map for the identification of mediastinum and hilar lymph nodes. *Thorac Cancer.* 2015;6:464-468.

**2.** Rusch VW, Asamura H, Watanabe H, et al. The IASLC lung cancer staging project: a proposal for a new international lymph node map in the forthcoming seventh edition of the TNM classification for lung cancer. *J Thorac Oncol.* 2009;4:568-577.

**3.** Yang F, Dong J, Wang X, Fu X, Zhang T. Non-small cell lung cancer: Spectral computed tomography quantitative parameters for preoperative diagnosis of metastatic lymph nodes. *Eur J Radiol.* 2017;89:129-135.

**4.** Zhang L, Fried DV, Fave XJ, Hunter LA, Yang J, Court LE. IBEX: an open infrastructure software platform to facilitate collaborative work in radiomics. *Med Phys.* 2015;42:1341-1353.

**5.** Sollini M, Cozzi L, Antunovic L, Chiti A, Kirienko M. PET Radiomics in NSCLC: state of the art and a proposal for harmonization of methodology. *Sci Rep.* 2017;7:358.

**6.** Lou Z, Xia B, Su J, et al. Effect of a stilbene glycoside-rich extract from Polygoni Multiflori Radix on experimental non-alcoholic fatty liver disease based on principal component and orthogonal partial least squares discriminant analysis. *Exp Ther Med.* 2017;14:4958-4966.
